# Significance of E-cadherin Gene Mutations in Patients With Hereditary Diffuse Gastric Cancer Syndrome: A Systematic Review

**DOI:** 10.7759/cureus.10406

**Published:** 2020-09-12

**Authors:** Harshit K Goud, Zainab Mehkari, Lubna Mohammed, Moiz Javed, Aldanah Althwanay, Farah Ahsan, Federico Oliveri, Ian H Rutkofsky

**Affiliations:** 1 Internal Medicine, California Institute of Behavioral Neurosciences & Psychology, Fairfield, USA; 2 Obstetrics & Gynaecology, California Institute of Behavioral Neurosciences & Psychology, Fairfield, USA; 3 Cardiology, California Institute of Behavioral Neurosciences & Psychology, Fairfield, USA; 4 Psychiatry, Neuroscience, California Institute of Behavioral Neurosciences & Psychology, Fairfield, USA

**Keywords:** cdh1, e-cadherin, hereditary gastric diffuse cancer, invasive lobular breast cancer, prophylactic gastrectomy, signet ring cell adenocarcinoma

## Abstract

Gastric cancer is the third-most fatal cancer in the world. Though over the years, we saw patients mostly with intestinal type accounting for the highest mortality rate, the recent rise of the diffuse form with germline E-cadherin (CDH1) mutations has added a whole new level of interest to study in detail about the association between CDH1 and diffuse gastric cancer (DGC). This introduced a set guideline formulated by Internal Gastric Cancer Linkage Consortium (IGCLC) for patients with family history of diffuse gastric cancer and invasive lobular breast cancer (ILBC). The analysis of this link was also important to set proper management protocol for patients who were CDH1 mutation carriers which now involves genetic counselling, endoscopic surveillance and screening and prophylactic total gastrectomy (PTG). The study was conducted in accordance to the ‘PRISMA guidelines for reporting systematic review and meta-analysis’. Peer-reviewed studies were included from the PubMed database and relevant articles were selected to be included in the study. Appropriate inclusion/exclusion criteria with free full text English articles were applied while selecting the articles. A total of 10 studies on review with different study populations showed that of the 42 patients who were diagnosed with diffuse gastric cancer, 88% of them showed a positive germline E-cadherin gene mutation and 100% of the CDH1 mutation carriers showed microscopic changes of signet ring cell adenocarcinoma of the stomach. The beneficial effects of PTG with better survival rates and low mortality rates has outweighed other treatment modalities. Laparoscopic approach has proved to be more useful and a safer approach for gastrectomy surgeries with better post-operative management. The need for prophylactic mastectomy is also increased in the recent times and thus this requires a new set of guidelines for ILBC patients with hereditary diffuse gastric cancer (HDGC) syndrome.

## Introduction and background

Gastric cancer, an aggressive form of cancer, has one of the highest incidences and mortality rates in the world. Incidence of gastric cancer (age 0-74) is the fifth highest in the world amongst both the sexes, being fourth highest among males and eight highest among females. Fatality is very imminent especially with advanced stages of gastric cancer, with the third highest mortality rate worldwide (age 0-74) (third highest among males and fifth highest among females) [1]. Gastric cancer can be divided into types, intestinal type and diffuse type [2]. The overall incidence and mortality rates of sporadic and intestinal type of gastric cancer have decreased in the recent years but an increase is seen in diffuse type of gastric carcinomas especially in the young age group of 20-50 years [2,3]. The most common type seen in the entire population is the sporadic, intestinal type of adenocarcinoma with its risk factors being the diet associated with it like eating spicy foods, alcohol use and the very notorious Helicobacter pylori infection. On the other hand if we study the diffuse gastric cancer (usually hereditary), whose incidence is on the rising trend in the recent years in the younger population, it takes a small proportion of the total gastric cancers in the world but usually has fatal outcomes in these patients because of its aggressive nature of infiltrating the mucosal wall of the stomach known as linitis plastica. The outcomes are fatal because of their late presentation of symptoms in their course and by then the spread engulfs major organ systems of the body [4].

The total proportion of the inherited form of gastric cancer is relatively low as compared to all other types, it is found to be between 1-3% and these are usually found out to be diffuse type of gastric carcinomas [5]. A close linkage between a cell-cell adhesion molecule, E-cadherin, and development of hereditary diffuse gastric carcinoma due to suppression and loss of heterozygosity by E-cadherin (CDH1) was found in 1998 in a New Zealand family. It was also established that most CDH1 mutations were found to be closely linked with familial history [6]. The establishment of this association between CDH1 and hereditary diffuse gastric cancer (HDGC) was a breakthrough which was needed for early identification of this genetic mutation in early prevention and management. Various articles and clinical guidelines were set for early diagnosis of these tumors who harbor these mutations and also adequate treatment protocols were set once they were found to have CDH1 mutation. One of the articles showed almost 64% of the signet ring cell carcinoma (SRCC) were found to be positive with CDH1 mutation [7].

Another close association of CDH1 mutation and invasive lobular breast cancer (ILBC) was found in the studies where risk of breast cancer was found in 39.9% of people and combined risk of 90% to develop diffuse gastric and ILBC by the age of 80 [8]. Further association of CDH1 mutation in patients with colorectal cancer was found and the suppression of CDH1 in pathogenesis of these tumors resulted in an opinion in the cancer society of a syndromic association between these tumors [8]. A criteria was set which keeps getting revised as and when new data arrives but the current criteria is as follows: one case of diffuse type of gastric cancer in a family with two or more cases of gastric cancer; diagnosis of diffuse gastric cancer before the age of 40; familial or personal history of invasive lobular breast cancer and diffuse gastric cancer if at least one person was diagnosed before the age of 50 [8,9]. A study assessing the risk of CDH1 mutations in patients with gastric and lobular breast carcinoma showed a risk percentage of 60-70% in males and around 80% in females for diffuse gastric cancer and the same study showed 40-50% of lobular breast cancer in women [10]. Thus people who fit into the above clinical criteria for hereditary diffuse gastric cancer syndrome undergo multiple endoscopies and biopsies and CDH1 mutations are studied in such people and in some cases prophylactic total gastrectomy (PTG) are performed halting the further progression of the CDH1 mutations in the cells thus improving the survival rates. In women, mammography screening is recommended to be done routinely to rule out any molecular or morphological alteration of the breast tissue [8].

This review article will deal with the diffuse type of hereditary gastric cancer in detail, the genetic associations of it, their relation to lobular breast cancer and colorectal cancer and also the management and interventions introduced in the recent years to stop the progress of such malignancy or to prevent it altogether.

## Review

Method

Review Protocol

The study was conducted following the Preferred Reporting Items for Systematic Reviews and Meta-Analysis (PRIMSA) guidelines for reviewing and recounting for systematic reviews and meta-analysis.

Literature Search

Peer-reviewed published articles were searched with the help of ‘PubMed’ database. The keywords used to find the desired articles for evaluating the significance of CDH1 (E-cadherin) mutation in cases with hereditary diffuse gastric cancer and invasive lobular breast cancer were as follows: CDH1, E-cadherin, gastric carcinoma, breast carcinoma, colorectal carcinoma, signet ring cell carcinoma - then combining the keywords for specific data and emphasis on the main subject - CDH1 and gastric carcinoma, CDH1 and colorectal carcinoma, CDH1 and breast carcinoma. The results shown by the PubMed database after typing in those keywords are as follows:

· CDH1 showed 2,571 peer-reviewed PubMed articles.

· E-cadherin showed 18,212 peer-reviewed PubMed articles.

· Gastric Carcinoma showed 13,863 peer-reviewed PubMed articles.

· Breast Carcinoma showed 111,780 peer-reviewed PubMed articles.

· Colorectal Carcinoma showed 65,522 peer-reviewed PubMed articles.

· Signet Ring Cell Carcinoma showed 1,094 peer-reviewed PubMed articles.

· On combining the keywords which were relevant for the current study, CDH1 and Gastric Carcinoma showed 127 peer-reviewed pubmed articles.

· CDH1 and Breast Carcinoma showed 390 peer-reviewed PubMed articles.

· CDH1 and Colorectal Carcinoma showed 149 peer-reviewed PubMed articles.

Inclusion/Exclusion Criteria

The inclusion protocol was formed for the study and they were as follows: all the peer-reviewed published articles irrespective of their study design and type were included in the study (case-reports, case series, reviews, systematic reviews, meta-analysis, cohort studies, clinical trials); ‘free-full’ text articles were only included in the study; published articles from 1998-2020 were included in the study; articles which were found only after typing the above keywords for the study were included in the study; articles which were written in English language were only selected. The articles which fulfilled the quality criteria were only selected. The rest of the articles which did not fulfil the above criteria were excluded from the study. All articles which consisted of the same population or the same case group were removed from the study.

Review Strategy

Peer-reviewed published articles included for the study were reviewed by the primary author after following all the PRISMA guidelines and fulfilling the inclusion/exclusion criteria. The review strategy was to find the articles which showed a relation between the CDH1 mutation and gastric and breast cancer as well as colorectal carcinoma. The main aim of the study was to establish the relevance of E-cadherin mutations seen in hereditary diffuse gastric cancer and invasive lobular breast cancer and how it affects the survival rate in these patients. The guidelines set for management and the prophylactic treatment available for patients is also discussed in detail in this study.

Results

**Figure 1:**
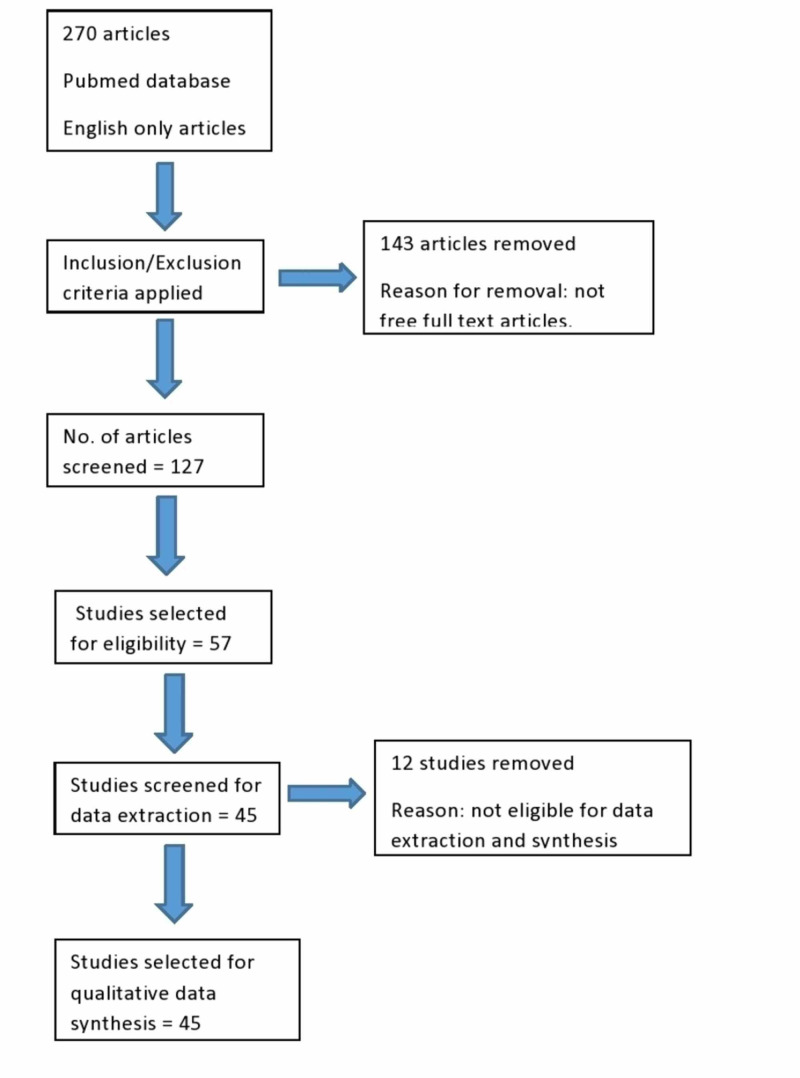
PRISMA flow diagram for study selection and data synthesis PRISMA: Preferred Reporting Items for Systematic Reviews and Meta-Analysis

The final study included a total of 57 papers of which 45 (consisting of case reports, observational studies and systematic reviews) were used for data extraction and synthesis, as shown in PRISMA flow diagram in Figure 1 [11].

The 12 studies which did not fulfil the quality appraisal were removed. The quality of the articles was assessed by appropriate quality assessment tools. The main focus of data extraction was to look for the significance of CDH1 mutation and HDGC syndrome. E-cadherin, a cell-cell adhesion molecule, on its downregulation is responsible for tumor growth and a close link between this CDH1 gene and HDGC is studied here. The studies found suggested that a CDH1 mutation carrier has an increased risk of developing diffuse gastric cancer and invasive lobular breast cancer in his/her life. The studies also focused the management protocol for such patients with treatment modalities being offered to either prevent or cure the disease. Prophylactic total gastrectomy (PTG) is the best option considered for such patients with a lifelong diet management to avoid nutritional deficiencies and other complications. Some studies included in the study showed the importance of prophylactic mastectomy for the carriers which is not currently in the guidelines. The role of non-CDH1 mutation causing HDGC has been introduced and the various DNA repair genes affected and malignancy caused is also studied.

Discussion

1. E-Cadherin Structure and Function

**Figure 2:**
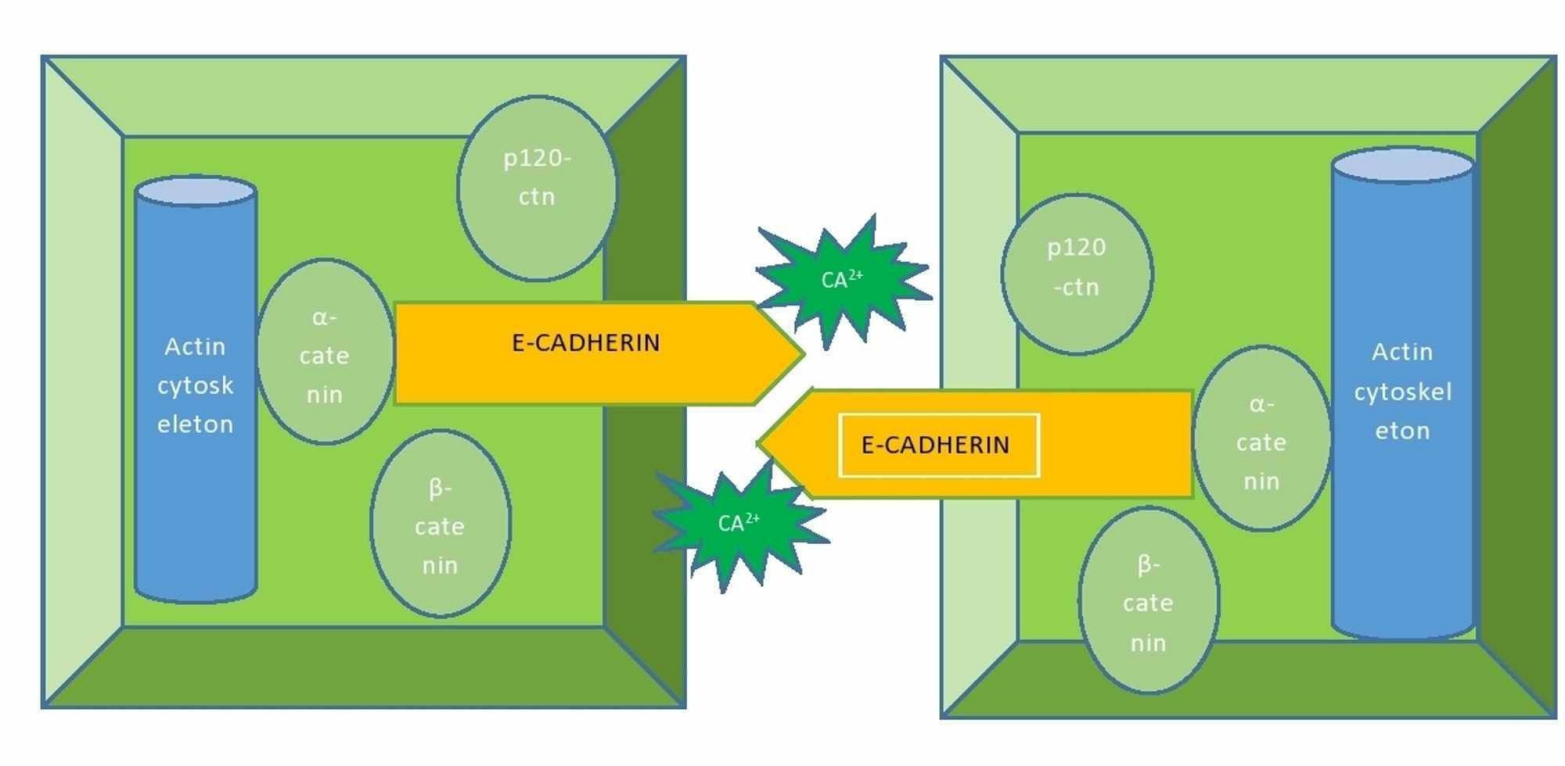
Mechanism of action of E-cadherin The cell-cell homeostasis maintained by the E-cadherin molecules with the help of the proteins and calcium ions. CA^2+^- Calcium ions.

E-cadherins are calcium dependent cell-cell adhesion transmembrane glycoproteins which are responsible for many structural cellular changes and also their development and their respective actions. They bind with various proteins intracellularly with the help of their cytoplasmic tail which interacts with α, β, γ catenins, p120ctn which further acts on the cytoskeleton structures, and brings about the morphological changes in the cell, as shown in Figure 2 [12]. Now, E-cadherin has an intracellular and an extracellular domain. The extracellular domain is where the calcium dependent E-cadherins interact with each other and the intracellular domain is where that interaction brings about the activation of further downstream signals in the cells by, as explained before, activation of the various catenin and p120ctn proteins [13]. The role of E-cadherin is the calcium dependent cell-cell interaction and thus maintaining tissue architecture but in malignancies, this interaction breaks and cell-cell homeostasis is imbalanced, so in simple terms, cancers are uncontrolled proliferation of tissue cells and one of the transmembrane glycoprotein, E-cadherin, is responsible to prevent that uncontrolled proliferation [14]. So when these cancer cells break the cell-cell binding interactions between E-cadherins, a vicious cycle of uncontrolled activation of the catenins and other proteins and their signaling pathways begin inside the cells which result in the multiplication of these cells finally resulting in uncontrolled growth of the tissue and thus a tumor, and thus this strong linkage between cancer and E-cadherin is what led us to believe that E-cadherin is a tumor suppressor gene and loss of function or missense mutations in these E-cadherin proteins brought about by cancer cells can cause in their loss of activity and thus leading to tumorigenesis [14]. A strong connection is also found in E-cadherin and metastasis of tumors which happens when the cell-cell linkage is lost and the bond is broken, the primary tumor has the ability to invade the surrounding tissue and travel to distant sites thus causing metastatic cancers [15]. Another protein associated with E-cadherin signaling other than catenins was found to be p120ctn. One of the studies showed that p120ctn is required for E-cadherins for strong cell-cell adhesions and when this protein is uncoupled from the E-cadherin, it destabilizes and the adhesion between the cells becomes weak and thus can result in malignancy [16]. The relation of E-cadherin mutations with invasive lobular breast carcinoma and gastric carcinoma was well established by various studies with loss of expression of E-cadherins on the apical luminal epithelial cells of the breast results in ILBC and their missense or in-frame deletions in development of HDGC proved their role in tumorigenesis and metastasis [17]. These mutations resulted in the intracellular activation of the above signaling pathways in the cells which led to tumor growth and invasion.

2. Hereditary Diffuse Cancer Syndrome: Testing Guidelines

Hereditary diffuse gastric cancer as said earlier has been found to have a close linkage to CDH1 mutation and associated with invasive lobular breast carcinoma, colorectal carcinoma and cleft lip/cleft palate. All the above-mentioned conditions were then brought under a single umbrella and called ‘hereditary diffuse gastric cancer syndrome’. The International Gastric Cancer Linkage Consortium (IGCLC) in the recent years have been updating their clinical guidelines for HDGC because of the increased incidence especially in the young. The most recent guidelines were updated in 2015, as can be seen in Table 1 [8].

**Table 1:** IGCLC criteria for HDGC syndrome DGC: Diffuse Gastric Cancer; LBC: Lobular Breast Cancer; IGCLC: International Gastric Cancer Linkage Consortium; HDGC: Hereditary Diffuse Gastric Cancer.

Absolute criteria	Relative criteria
Family with two cases of gastric cancer with one confirmed case of diffuse gastric cancer	Family with more than two cases of LBC under 50 years of age or history of bilateral LBC.
Family with one confirmed case of diffuse gastric cancer under 40 years of age	Cleft lip/palate, personal or family history along with DGC.
A personal or family history of diffuse gastric cancer and lobular breast cancer under 50 years of age	Histopathology showing pagetoid signet ring cells spread or in situ signet ring cells.

If a person satisfies the clinical criteria, they go for CDH1 testing if they consent, if the test results come back negative, then the patient is sent to study for clinical research and to study the other factors responsible for development of gastric cancer and if the test result comes back positive, then a team of doctors decide the further management for the patient [8]. CDH1 positive patients undergo routine endoscopies and biopsies are taken to study the gastric tissue and if the biopsy demonstrates signet ring cell changes or in situ carcinoma, they are advised for prophylactic gastrectomy [8].

3. CDH1 Surveillance in-

3A. Hereditary Diffuse Gastric Cancer

CDH1 is a tumor suppressor gene, so any mutation resulting in its loss of function can cause derangements in the basic cellular structure of the tissues resulting in a neoplasm or an irregular growth which can later develop into a malignancy. It is related to many tumor pathologies but what makes it more interesting here is its close association with HDGC proven recently in various studies. Loss of function mutation or missense mutation in the CDH1 gene has resulted in HDGC more often than not. And the recent decline in the non-cardia gastric cancers and increase rate of DGC in the young population < 50 has shifted the focus of researchers to study in detail the link between CDH1 and HDGC. Here in the current study, as shown in Table 2, we have reviewed 10 clinical case studies of various families with either personal or family history of DGC who underwent genetic testing for CDH1 mutation.

**Table 2:** CDH1 mutation and HDGC association shown in case reports DGC: Diffuse Gastric Cancer; CDH1: Cadherin-1 or E-cadherin; SRCC: Signet Ring Cell Cancer; M: Male; F: Female; HDGC: Hereditary Diffuse Gastric Cancer.

S. no.	Author	Study population	M:F ratio	Germline CDH1 mutation	DGC positive	Tumor histology
1	Zylberberg et al., 2018 [18]	22	8:5	13	9	SRCC
2	Yelskaya et al., 2016 [19]	3	3:0	3	3	SRCC
3	Caggiari et al., 2018 [20]	2	2:0	2	1	SRCC
4	Shepard et al., 2016 [21]	3	0:1; Rest unknown	1	3	SRCC
5	Gjyshi et al., 2018 [22]	6	3:3	3	3	SRCC
6	Campos et al., 2015 [23]	5	2:1; Rest unknown	1	3	SRCC
7	Norton et al., 2007 [24]	6	2:4	6	6	SRCC
8	Black et al., 2014 [25]	4	2:2	4	4	SRCC
9	Herraiz et al., 2012 [26]	5	2:3	2	4	SRCC
10	Keller et al., 1999 [27]	7	Unknown	2	6	SRCC
	Total	63 patients	24:19	37 patients with CDH1 mutations	42 positive for DGC	

Out of 63 people in the study who were suspected with DGC based on their personal and family history, 67% (42 patients) came back positive for DGC and out of the 67% of positive DGC cases, 88% (37 patients) were found to have a positive CDH1 mutation in them and their family. So 58% of the total number of people in the study were found to be CDH1 gene mutation positive. Males were found to be more prone to develop HDGC than females in the study. All the patients in the study were advised to undergo a routine endoscopy regularly and periodic biopsies to check for any changes in their tissue architecture. All the 42 patients were found to have signet ring cell adenocarcinoma on tissue morphology. Some of the mutations studied in the patients were found to have either missense mutation or frameshift mutation, missense mutation being the commonest [18-27]. A study was conducted by Brooks-Wilson et al., which included 43 different families, 42 positive for DGC and the other one being intestinal type and in the study, CDH1 mutations were found in more than 30% of the families and 12 of those families showed familial history of multiple cases of gastric and lobular breast cancer with an autosomal dominant inheritance pattern of these mutations and almost 50% of those mutations were found to have a germline truncating CDH1 mutations [28]. The autosomal dominant inheritance pattern of these mutations requires a second-hit for cancer progression and this second hit usually in a germline CDH1 mutation was found to be due to promoter hyper methylation [29]. In a study conducted among 183 patients who met the IGCLC clinical criteria for HDGC, almost 20% of the cases showed a definite CDH1 mutations and the lifetime risk of developing HDGC was found to be between 60-80% in males and almost 60% in females and the lifetime risk of developing ILBC was found to be more than 42% with the same CDH1 mutations [10]. The rising trend of increased HDGC cases can also be proven from the fact that countries like Japan, China, Korea and other south east Asian countries, where gastric cancer is most commonly attributed to the Helicobacter pylori infection or a high-spice diet, are seeing an increase in number of HDGC predominantly due to CDH1 mutations. One such case was reported in Korea in 2014 where a 44-year-old male, who on genetic testing presented a family history of gastric cancer with no symptoms, visited the clinic for genetic testing and then later on was found to have a CDH1 mutation, with this being one of the first cases reported in the region with such history and mutation during the time and so was kept on regular endoscopic surveillance [30].

The diagnostic modality to confirm the diagnosis for these tumors is an important aspect to deal with because these tumors have a discrete foci, usually very small and because their highly infiltrative properties do not appear on the routine endoscopic surveillance easily and that is why biopsy is gold standard for confirming the diagnosis of HDGC in patients, because it not only helps us confirm the diagnosis but also helps in assessing the tumor staging and classification [8]. More and more studies and trials are being conducted for different treatment modalities for prevention and treatment of CDH1 mutation carriers which requires a team of doctors including surgeons, oncologists, geneticists, psychiatrists and nutritionists who help the patient decide for the next best step in their treatment [8].

In the studies mentioned in the review, all of them underwent a biopsy to confirm their diagnosis and the patients who were found to be carriers after a thorough family genetic testing were advised for a triennial endoscopic surveillance to check for any tumor growth [8].

3B. Invasive Lobular Breast Carcinoma

ILBC is not a common type of breast carcinoma, seen in only about 10-20% of the population with rest being the ductal carcinoma type [31]. Once diagnosed, the prognosis is usually good with target chemotherapies for HER2neu receptors. But when it is a due to the E-cadherin dysfunction in the cells, which helps in cellular binding, the cells become non-cohesive and the tissue loses its basic structure resulting in metastasis and unregulated growth of the cells [31]. As in HDGC, the second-hit resulting in the tumor growth is most commonly due to promoter methylation with one case series showing that in 77% of their study population, the main cause of inactivation of the tumor suppressor gene CDH1 was promoter methylation [32]. No ductal carcinomas till date have been associated with a CDH1 loss of expression though they are present in decreased amounts in such cases but a direct relation or link has not been established yet [33]. A study by Keller et al. in 1999 was one of the first studies to establish a link between breast carcinoma, diffuse gastric carcinoma and CDH1 germline mutation where a basepair (bp) deletion on exon 3 was detected in a patient suspected with diffuse gastric carcinoma - with patient having a strong young age family history of DGC and the mother of the patient was diagnosed with both diffuse gastric cancer and lobular breast cancer [27]. When exon 3 in both the patients was analyzed, the same deletion was found on chromosome 16q22.1, thus establishing a germline mutation between the two patients [27]. A study was conducted among 38 families to establish the germline CDH1 mutation link in cases with diffuse gastric cancer where four families from Newfoundland in the study were found to have a common haplotype mutation which causes both DGC and ILBC and two families among the four had later on developed lobular breast cancer. The risk assessment for development of DGC by the age of 75 years in males was found to be around 40% to 63%, and in females the development of lobular breast carcinoma was found to be 52% in the study [34]. Suriano et al. in 2005 conducted a CDH1 genetic mutational analysis, where a family history of lobular breast carcinoma was found with a similar DGC causing CDH1 mutation and another individual who presented with both DGC and ILBC with the same mutation [35]. Xie et al. published a case series where he studied two families which presented with six ILBC but no history of DGC, showing similar mutational characteristics of inactivation of CDH1 gene shown by other studies, and Corso et al. in 2016 also published an article showing similar findings [36,37].

3C. Other Associated Malignancies and Conditions

A very rare and peculiar case was published in 2013 by Hamilton et al. where an appendectomy performed in an appendicitis suspected patient showed signet ring cell morphology on histopathological examination which correlates with her CDH1 mutation carrier status. She also underwent prophylactic gastrectomy because of carrier status [38]. No other similar case was published after this. Though the available data for CDH1 association with colorectal cancer is scarce, screening of family members for colon cancer should begin by the age of 40 especially when a family has previous case of colon cancer [39]. Pancreatic ductal carcinoma in an HDGC patient with the same germline mutational event has not been established yet but there is found to be a sporadic loss of expression of CDH1 gene. More studies focusing on establishing a relation between these two might help us understand more about HDGC syndrome since pancreatic ductal carcinoma can be a very aggressive tumor [40]. The most interesting association is found between Cleft Lip/Cleft Palate (CL/CP) cases in patients who are CDH1 mutation carriers and patients who have developed DGC in the future. This is classically presented in a case report of a 17-year-old CL/CP girl from SE Asia who was getting investigated for her iron deficiency anemia (IDA) symptoms. On the gastric biopsy, signet ring cells were found which had crossed the basement membrane but on endoscopy looked normal. Genetic testing was done and she was found to have a CDH1 mutation thus confirming the diagnosis of DGC. Various studies conducted in Denmark and France also found similar results where 7% of the test population of CDH1 mutation carriers were found to have CL/CP. This has increased the probability of finding CDH1 mutation carriers in patients suffering from CL/CP and thus if diagnosed, a future risk of DGC can be averted [41-44].

4. Treatment Guidelines: Surveillance and Prophylactic Surgeries

**Figure 3:**
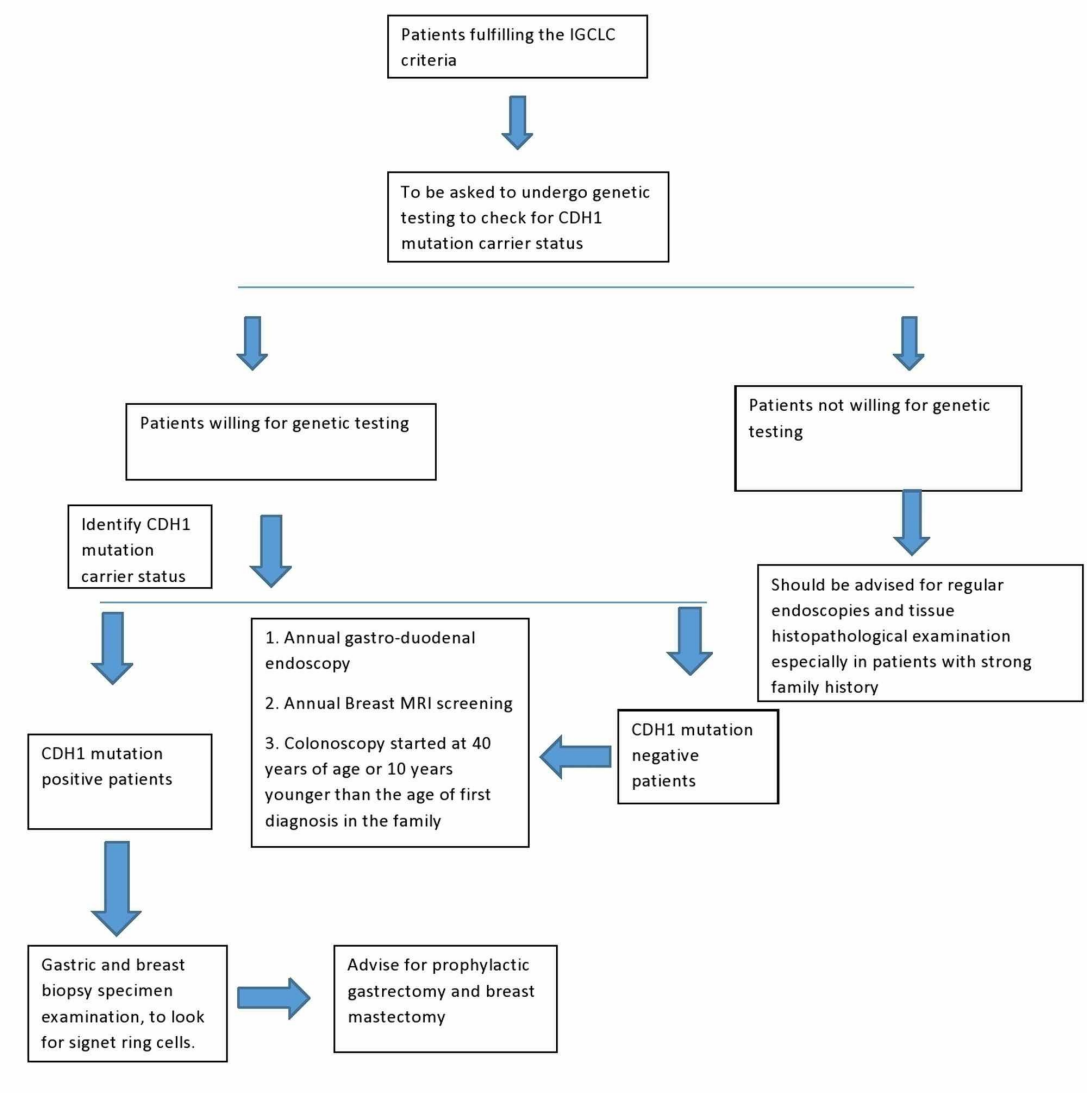
Screening and management of patients with CDH1 mutation gene IGCLC: International Gastric Cancer Linkage Consortium; CDH1: Cadherin-1 or E-cadherin; MRI: Magnetic Resonance Imaging.

As the incidence of HDGC increased in the world, guidelines for its management was deemed important. IGCLC sets the new guidelines every few years whenever there is a significant breakthrough or discovery of a new diagnostic feature which helps in better understanding and proper management of HDGC. First the criteria were set for the people who should undergo testing for HDGC risk assessment. And the patients who fulfil the criteria are asked for periodic surveillance and screening and appropriate treatment by following the standard protocols set by IGCLC, as shown in Figure 3 [8].

Genetic counselling of these patients is important to assess the current and the future risk for HDGC, as can be seen in Table 3. This is conducted by the geneticist who has advanced expertise in the area [8]. A pedigree chart is made to assess germline hereditary cancers presented by the patient’s family. And to complete the process for genetic testing of the patient, an informed consent is must [8]. For the patients who are not willing for genetic testing must be asked to come for regular visits if they develop any complaints and also for regular endoscopies to look for gastric mucosal changes [8].

**Table 3:** Risk analysis in CDH1 mutation patients DGC: Diffuse Gastric Cancer; ILBC: Invasive Lobular Breast Cancer; CDH1: Cadherin-1 or E-cadherin.

Risk assessment in CDH1 mutation carriers	Men (by 75-80 years of age)	Women (by 75-80 years of age)
DGC	60-80%	40-70%
ILBC	-	20-70%

5. Endoscopic Surveillance

A multi-disciplinary team is a must in the hospital setting for HDGC cases. The team consists of the geneticists, surgeons, radiologists, physician, oncologists, psychiatrist, nutritionists and other necessary members from their respective fields [8].

Patients who are found to be CDH1 mutation positive, are asked to undergo endoscopic surveillance of the gastric tissue to look for any morphological changes seen in the tissue. If an area is suspected, biopsy must be taken and then sent for histopathological examination to look for the pagetoid spreading of the characteristic signet ring cells [8]. Fitzgerald et al. showed that even if the tissue looks normal on endoscopy, mandatory biopsies must be taken from the pre-pyloric area, antrum, body, cardia, fundus and transition zone. Cambridge protocol dictates that at least 30 biopsies should be taken from the stomach [45]. Along with endoscopy, a high-resolution multi-detector computed tomography (CT) and endoscopic ultrasound (EUS) must also be done to investigate the stomach wall layers to check for any infiltration of malignant tumor. Patients who are found to be CDH1 negative, but show a strong family history of HDGC must undergo annual endoscopy procedure to look for gastric tissue variations, regular breast clinical examination and mammography though not sensitive and annual MRI breast screening [8].

*6*.* Operative Management*

Patients who are found to be CDH1 positive are advised to undergo ‘prophylactic gastrectomy’. The patient must be provided adequate information about the procedure and the lifestyle changes required post-operatively. The multi-disciplinary team (MDT) plays an active role in the management of the patient both pre-operatively and post-operatively. The patients who are CDH1 positive and also develop symptoms and when on examination are found to have an advanced stage of disease, the recovery rate even after surgery is not more than 10% and the five-year survival rate not more than 30% in such patients and so a properly informed decision must be made by the patient to undergo this procedure [8]. The operation most commonly performed is the ‘Total Gastrectomy with Roux-en-Y reconstruction anastomoses'. Frozen section should be performed on the resected specimen to check for the proximal margins which should contain any gastric mucosa and for ILBC, prophylactic mastectomy is routinely not done, unless the patients develop symptoms and the there is no sufficient literature available for the same [8]. An article published recently though showed that patients who are CDH1 mutation carriers and also are at an increased risk of developing ILBC in the future because of a very strong family history, should undergo bilateral prophylactic mastectomy along with reconstruction [46]. Though not included in the guidelines yet but this is an area to think about the management protocol for high-risk ILBC families.

The newer method of approach for gastrectomy is through laparoscopy. Several studies showed better outcomes and patient-willingness for laparoscopic approach to surgery. One study performed six laparoscopic gastrectomy on CDH1 mutation carrier patients and concluded with increased patient compliance for low-risk and safe laparoscopic approach. Histopathological examination of all six specimens showed invasive signet ring cell changes [47]. A study conducted in 2007 was one of the first ones to have a laparoscopic approach for total gastrectomy with favorable results [48]. Another study was conducted on 11 CDH1 mutation carrier patients who underwent laparoscopic total gastrectomy with jejunal pouch reconstruction and the similar results were obtained. Interestingly, when tested for histopathology, more than 80% of the resected specimen were found to have invasive SRCC [49]. The occurrence of post-operative symptoms such as leakage, blood loss, wound rupture is also low in laparoscopic procedures [48,49].

Following all the procedures, the need for dietician becomes very important in the patient’s life. Malabsorption and nutritional and vitamin deficiency are very common and a proper diet must be followed. Dumping syndrome, a very common post-operative complication after gastrectomy because of rapid passage of food particles from esophagus to the small bowel, can be improved by following necessary restrictions in dietary habits [8]. Future incidence of strictures, difficulty in eating and nutritional anemia is common in such patients and so a proper work-up with addition of multi-vitamin supplements must be continued lifelong in such patients but usually after the first year of surgery; almost all patients show complete signs of recovery and thus can slowly begin their normal life activities with dietary restrictions [8].

7. Others

A study was conducted by Fewings et al. where they studied different genes responsible for HDGC in families where there were no CDH1 mutations and this was an important study since the data for non-CDH1 carrier HDGC management is not sufficient enough [50]. Depicted in Table 4 are the genes which were found to be associated with no pathological E-cadherin variant with most of these being DNA repair genes where a single protein was affected resulting in a germline mutation and the gene which was best recognized to play a significant role in development of HDGC in non-CDH1 mutational carriers was found to be PALB2 [50]. The age of diagnosis for these tumors with the said gene mutations ranged from 22-83 years with 27% of the families studied were found to have non-CDH1 gene mutations and were diagnosed with HDGC [50].

**Table 4:** Genes and their associated malignancies PALB2- Partner And Localizer of BRCA2 (Breast Cancer Gene) RECQL5- ATP dependent DNA Helicase Q5 MSH2- DNA Mismatch Repair protein ATR- Ataxia Telangiectasia and Rad3 related protein NBN- Nibrin protein

Identified gene	Associated malignancies
PALB2	Breast, lung, laryngeal, gastric and diffuse gastric cancer
RECQL5	Breast and gastric cancer
MSH2	Diffuse gastric cancer
ATR	Diffuse gastric cancer
NBN	Diffuse gastric cancer

8. Limitations and Future Considerations

One of the main limitations of the study was including free full-text articles and leaving out many articles which were relevant but not available for free. The systematic review conducted for HDGC syndrome established the relationship between the CDH1 mutations and diffuse gastric cancer. Furthermore, observational studies must be conducted to help IGCLC in revising the management guidelines. There are no criteria set as of now for HDGC in non-CDH1 mutation carriers. This is where the observational studies will help us understand more about non-CDH1 carriers developing diffuse gastric cancer. The study was conducted in a population specific for DGC, so if a large-scale study happens in the future where a set of whole population can be brought under and studied will help us understand the true incidence of DGC in the population and also a higher significance if found can be established between CDH1 gene mutation and DGC. A large-scale study involving various experienced researchers and doctors from various fields must also be conducted to extract more information about the CDH1 pathogenic variants causing malignancies which have not been yet grouped into HDGC syndrome. A study is being conducted by the IGCLC to update the guidelines and its results will be published soon.

## Conclusions

The role of early screening and surveillance in CDH1 mutation carrier families cannot be stressed enough. With the current study showing a strong association between CDH1 and HDGC with 88% of DGC patients positive for CDH1 mutations and almost all of them with positive signet ring cell changes on microscopy on screening, awareness for an early preventive or curative approach is needed. The treatment modalities currently recommended by IGCLC are for a prophylactic total gastrectomy in the carriers. With the recent studies showing increased success and favorable results for laparoscopic approach for total gastrectomy, the operative management of these patients will see further changes in the future and move from open to laparoscopic gastrectomy approach. The current study also established a relation between ILBC and HDGC, where before the data was considered scarce to associate a link between them. The need for prophylactic mastectomy in these patients is a must to prevent a future risk of malignancy. Surveillance and screening in high-risk individuals is a must in CDH1 carriers. The role of multi-disciplinary team in managing CDH1 mutation positive patients is extensive. Proper management, rehabilitation and prevention of gastric malignancy in the patient should be the main goal.
